# Author Correction: Sustained antidepressant effect of ketamine through NMDAR trapping in the LHb

**DOI:** 10.1038/s41586-023-06814-x

**Published:** 2023-11-06

**Authors:** Shuangshuang Ma, Min Chen, Yihao Jiang, Xinkuan Xiang, Shiqi Wang, Zuohang Wu, Shuo Li, Yihui Cui, Junying Wang, Yanqing Zhu, Yan Zhang, Huan Ma, Shumin Duan, Haohong Li, Yan Yang, Christopher J. Lingle, Hailan Hu

**Affiliations:** 1 Department of Psychiatry and International Institutes of Medicine, The Fourth Affiliated Hospital, Zhejiang University School of Medicine, Yiwu, China; 2 Nanhu Brain–Computer Interface Institute, MOE Frontier Science Center for Brain Science and Brain–Machine Integration, State Key Laboratory of Brain–Machine Intelligence, New Cornerstone Science Laboratory, Zhejiang University, Hangzhou, China; 3 Department of Affiliated Mental Health Center and Hangzhou Seventh People’s Hospital and School of Brain Science and Brain Medicine, Zhejiang University School of Medicine, Hangzhou, China; 4 Department of Anesthesiology, Washington University School of Medicine, St Louis, MO USA

**Keywords:** Depression, Ion channels in the nervous system, Emotion

Correction to: *Nature* 10.1038/s41586-023-06624-1 Published online 18 October 2023

In the version of the article initially published, the sentence “C.J.L. is supported in part by R35GM118114 from NIGMS” was missing from the Acknowledgements section and has now been inserted in the HTML and PDF versions of the article.

